# Frailty trajectories to identify end of life: a longitudinal population-based study

**DOI:** 10.1186/s12916-018-1148-x

**Published:** 2018-09-21

**Authors:** Daniel Stow, Fiona E. Matthews, Barbara Hanratty

**Affiliations:** Institute of Health and Society, Newcastle University, Newcastle upon Tyne, NE2 4AX UK

**Keywords:** Frailty, Geriatrics, Palliative care, Primary care, End of life

## Abstract

**Background:**

Timely recognition of the end of life allows patients to discuss preferences and make advance plans, and clinicians to introduce appropriate care. We examined changes in frailty over 1 year, with the aim of identifying trajectories that could indicate where an individual is at increased risk of all-cause mortality and may require palliative care.

**Methods:**

Electronic health records from 13,149 adults (cases) age 75 and over who died during a 1-year period (1 January 2015 to 1 January 2016) were age, sex and general practice matched to 13,149 individuals with no record of death over the same period (controls). Monthly frailty scores were obtained for 1 year prior to death for cases, and from 1 January 2015 to 1 January 2016 for controls using the electronic frailty index (eFI; a cumulative deficit measure of frailty, available in most English primary care electronic health records, and ranging in value from 0 to 1). Latent growth mixture models were used to investigate longitudinal patterns of change and associated impact on mortality. Cases were reweighted to the population level for tests of diagnostic accuracy.

**Results:**

Three distinct frailty trajectories were identified. Rapidly rising frailty (initial increase of 0.022 eFI per month before slowing from a baseline eFI of 0.21) was associated with a 180% increase in mortality (OR 2.84, 95% CI 2.34–3.45) for 2.2% of the sample. Moderately increasing frailty (eFI increase of 0.007 per month, with baseline of 0.26) was associated with a 65% increase in mortality (OR 1.65, 95% CI 1.54–1.76) for 21.2% of the sample. The largest (76.6%) class was stable frailty (eFI increase of 0.001 from a baseline of 0.26). When cases were reweighted to population level, rapidly rising frailty had 99.1% specificity and 3.2% sensitivity (positive predictive value 19.8%, negative predictive value 93.3%) for predicting individual risk of mortality.

**Conclusions:**

People aged over 75 with frailty who are at highest risk of death have a distinctive frailty trajectory in the last 12 months of life, with a rapid initial rise from a low baseline, followed by a plateau. Routine measurement of frailty can be useful to support clinicians to identify people with frailty who are potential candidates for palliative care, and allow time for intervention.

**Electronic supplementary material:**

The online version of this article (10.1186/s12916-018-1148-x) contains supplementary material, which is available to authorized users.

## Background

An increasing number of older people are now dying with a diagnosis of frailty. In high-income countries, the estimated prevalence of frailty is 11% for people aged over 65 years, rising to 25–50% for people over 85 [1]. Frailty is characterised by an accumulation of deficits and reduced strength, endurance and physiological function [2, 3]. It is associated with a range of adverse outcomes, including falls, delirium, disability and mortality [4–8].

Timely recognition of the end-of-life phase is fundamental to the provision of palliative care, as it allows clinicians to introduce generalist or specialist services, to discuss preferences and make advance plans [9–11]. This may be particularly challenging for patients with frailty, where trajectories of decline are gradual and slow, and patients may not have a recognised life-limiting diagnosis [12, 13]. Despite calls for greater awareness of the benefits of palliative care for people with frailty [14], recognition remains incomplete [15].

Since 2017, general practitioners (GPs) in England have been required to identify and review patients with moderate and severe frailty using an appropriate tool. One of the instruments recommended by NHS England is the electronic frailty index (eFI) [16, 17]. The eFI is a cumulative deficit measure of frailty that uses over 2000 Read codes to calculate a frailty score based on a variety of symptoms, diagnoses and observations recorded in primary care electronic health records [5]. It is available in GP software systems in the UK, and can be calculated automatically from patients’ records at any time. Previous research has shown that frailty measured by the eFI at a single time point can predict mortality at a population level [17], but it is not effective at identifying individuals at short-term risk of all-cause mortality before this would be clinically apparent [18]. Frailty is associated with increasing age, but there is also wide inter-individual variation in how deficits are accumulated over time, and studies have demonstrated different longitudinal trajectories of frailty [19, 20]. However, to date, few studies have examined the association between longitudinal change in frailty and mortality. Where studies have been carried out, they have produced population level results with limited clinical application. For example, recent analysis of data from a US cohort identified distinct frailty trajectories and their associated mortality risk, using scores calculated at annual intervals over 8 years [21].

In this study, we used eFI measured at monthly intervals to examine longitudinal change in frailty in the year before death. The aim was to determine if changes in frailty measured by the eFI could be useful in primary care to indicate increased risk of dying and the need to consider palliative care.

## Methods

### Setting

The study population was sampled by ResearchOne from their health and care research database, containing de-identified clinical and administrative data from approximately six million active electronic healthcare records. ResearchOne extracts anonymised data from the SystmOne clinical information system, which is used in over 35% (2500) of general practices in England, and holds records on approximately half of the UK population. Primary care professionals use SystmOne to record their consultations (including patient histories, clinical observations, diagnoses, treatments and referrals) with free text and the Read code classification system.

### Participants

ResearchOne identified records of individuals (cases) aged 75 years and over who died between 1 January 2015 and 1 January 2016. A comparison group (controls) was constructed by identifying patients matched 1:1 to cases by age, sex and practice location, but with no record of death between 1 January 2015 and 1 January 2016. Patients without eFI scores, those with records available for fewer than 6 months, and those for whom cause of death was classified as an external cause of mortality (accidents, suicides, murders according to International Classification of Diseases codes version 10) were excluded.

### Study design

In this longitudinal study, the relationship between mortality and frailty was determined using eFI scores generated at monthly intervals for 1 year prior to recorded month of death for cases, and 1 year prior to 1 January 2016 for matched controls. Controls were matched at age of death of the case (between 1 January 2015 and 1 January 2016), and sampled on 1 January 2016, when they were known to have survived; hence, controls were, on average, 6 months older than the cases at time of measurement. Approval for this study was granted by the ResearchOne ethical review panel, with oversight from the UK NHS Health Research Authority and the UK Government Health and Social Care Information Centre Confidentiality Advisory Group. ResearchOne was approved by the UK NHS National Research Ethics Service (11/NE/0184).

### Measurements

The eFI is a cumulative deficit measure of frailty that calculates a frailty score based on 36 deficits, drawn from a pool of 2000 clinical Read codes for symptoms, signs, diseases, disabilities and abnormal laboratory test values. An individual’s eFI score is calculated by dividing the number of deficits present by the total possible to create a score between zero (no deficits) and one (36 deficits) [17]. In this study, eFI was calculated automatically by ResearchOne at monthly intervals for 1 year, based on the information contained in each participant’s clinical record.

### Analysis

Latent growth curve models were used to examine the shape of change in eFI and the relationship between eFI trajectory and age and sex (age mean centred and female as the referent categories). Latent class growth mixture models were used to examine the potential for subgroups (classes) of the population to have different trajectories of change in eFI. The optimum number of classes was determined using sample size-adjusted Bayesian information criterion (BIC) and model entropy. The trajectories estimated from the optimum latent class growth mixture model were used to examine the association between class membership and mortality (case/control status) as a distal outcome [22]. The longitudinal models were formulated using months as the time variable, with month 0 as baseline and month 12 at death/study end. All longitudinal models were run in Mplus version 8 (Muthén & Muthén, Los Angeles, CA, USA). To estimate the proportions of the population in each of the trajectory classes, adjusting for the study design (sample of controls), the posterior probabilities for being in each class were reweighted by the population estimates from Office of National Statistics life tables. Weighted class sizes and the association between class membership and mortality were calculated using the Survey package (Lumley, 2017) in R version 3.4.2 (R Core team, Vienna, Austria).

## Results

This study analysed information on 26,298 individuals (13,149 cases and 13,149 controls), 14,620 (55.6%) of whom were female and 11,678 (44.4%) were male. The mean age at death for cases was 85.14 (SD 5.98) years with a pseudo age of 85.66 (SD 5.98) years for controls, as expected by design. Age-adjusted mean eFI at baseline was significantly higher (*p* < 0.001) for cases (eFI = 0.27, 9.8 deficits) than for controls (eFI = 0.23, 8.3 deficits), as expected from previous work [17, 18], and mean eFI for females (eFI = 0.23, 8.3 deficits) was significantly higher (*p* < 0.001) at baseline than for males (eFI = 0.22, 8.0 deficits) when adjusted for age and case/control status. Table 1 shows the demographic characteristics of the study sample.

**Table 1:** Demographic characteristics of study participants at baseline

	Case		Control	
*n*	13,149		13,149	
Female (*n*, %)	7310	55.59	7310	55.59
Male (*n*, %)	5839	44.41	5839	44.41
Age, years (mean, SD)	85.14	5.98	85.66	5.98
Female age, years (mean, SD)	86.15	6.11	86.68	6.12
Male age, years (mean, SD)	83.87	5.55	84.38	5.54
eFI	27.05		23.27	
Female eFI	28.08		24.61	
Male eFI	25.76		21.59	
eFI (age adjusted)	27.18		23.13	
Female eFI (age adjusted)	27.87		23.84	
Male eFI (age adjusted)	26.30		22.27	

The latent growth curve model that best described the shape of frailty change over the study period included freely estimated intercepts, slopes and a quadratic polynomial term for time (see Additional file 1: Table S1 for parameter descriptions and model fit statistics). The estimated mean trajectory of eFI increased by 0.002 (95% CI 0.001–0.002, 0.1 deficits) per month (at study baseline) from a mean of 0.252 (95% CI 0.251–0.253, 9.1 deficits). The quadratic term was small (eFI = 0.00003, 95% CI 0.00003–0.00004) but improved the model fit and was retained to allow flexibility in class structures during the mixture modelling phase. Large variance in the intercept (eFI = 0.128) and slope (eFI = 0.278) was observed, suggesting individual heterogeneity in trajectories. There was some evidence that higher baseline frailty was related to slower frailty increase over time (intercept slope covariance − 0.109, *p* < 0.006).

The quadratic model was extended to investigate the impact of age and sex on the trajectories (Table 2 and Additional file 1: Table S2 for model fit and parameter descriptions). In the full model (Additional file 1: Table S2), eFI at study baseline increased by 0.0047 (95% CI 0.0044–0.0049, 0.2 deficits; *p* < 0.001) for each year of age, and men had a mean eFI of 0.0016 (95% CI 0.00133–0.00187, 0.6 deficits; *p* < 0.001) lower than women. Because there was little evidence that sex or age influenced the trajectory of frailty over time (for each year of age, eFI increased by 0.00002 per month, 95% CI 0.00001–0.00003, < 0.001 deficits, *p* = 0.003, all other coefficients *p* > 0.05), the model regressing the intercept only on age and sex, including slopes and quadratic terms (Table 2), was carried forward to investigate potential latent class trajectories.

**Table 2:** Summary of best fitting models for shape of change, the impact of study covariates and class descriptions

Model	Latent growth model: Intercept variance free, slope variance free, quadratic term added		Previous model plus study design covariates age and sex, intercept regressed on age and sex			Previous model specified as a latent growth mixture model with three classes	
Log likelihood	− 530,331.072		−529,340.060			− 519,497.615	
Adjusted BIC	1,060,816.13		1,058,848.110			1,039,247.203	
	Estimate^a^	95% CI	Estimate^a^	95% CI		Estimate^a^	95% CI
Intercept	25.185	(25.048 to 25.322)	25.893	(25.715 to 26.071)	class 1 ‘stable class’ (*n* = 20,144, 76.6%)	25.959	(25.763 to 26.155)
Slope	0.151	(0.144 to 0.158)	0.151	(0.144 to 0.158)		− 0.080	(− 0.085 to – 0.074)
Quadratic	0.003	(0.003 to 0.004)	0.003	(0.003 to 0.004)		0.015	(0.015 to 0.015)
Age on intercept	–	–	0.467	(0.444 to 0.489)		0.466	(0.444 to 0.489)
Sex on intercept	–	–	− 1.594	(− 1.864 to – 1.325)		− 1.598	(− 1.866 to – 1.329)
Intercept					class 2 ‘moderately increasing class’ (*n* = 5572, 21.2%)	26.232	(25.907 to 26.558)
Slope						0.802	(0.765 to 0.838)
Quadratic						− 0.029	(− 0.032 to – 0.027)
Age on intercept						0.466	(0.444 to 0.489)
Sex on intercept						− 1.598	(− 1.866 to – 1.329)
Intercept					class 3 ‘rapidly rising class’ (*n* = 582, 2.2%)	20.583	(19.387 to 21.779)
Slope						2.294	(2.115 to 2.472)
Quadratic mean						− 0.102	(− 0.114 to – 0.09)
Age on intercept						0.466	(0.444 to 0.489)
Sex on intercept						− 1.598	(− 1.866 to – 1.329)

^a^ Models used eFI multiplied by 100 to aid estimation and interpretation. Text in results refers to eFI in original units

A three-class latent growth mixture model model (Table 2) was chosen as the most parsimonious with good fit and clinically relevant classes (Additional file 1: Table S3). The most common ‘stable’ class contained 76.6% of the sample and showed little change in frailty over time, with eFI increasing by 0.001 per month (0.04 deficits, ‘stable’ class) from a baseline eFI of 0.260 (9.4 deficits). A ‘moderate growth’ class containing 21.2% of the sample demonstrated an eFI increase of 0.0045 (0.2 deficits) per month from a slightly higher baseline eFI of 0.262 (9.4 deficits), and a small ‘rapidly rising’ class containing 2.2% of the sample showed a sharp, distinct increase in eFI of 0.01 per month (0.4 deficits) on average over the year from a lower baseline eFI of 0.206 (7.4 deficits). The majority of individuals in this class accrued deficits quickly, with a 0.02 eFI increase per month over the first 3 months (s = 0.023, 95% CI 0.021–0.025), but the quadratic component (q = − 0.001, 95% CI – 0.001 to – 0.001) indicates that they then experienced a period of relative stability; this group had an average increase in eFI over 1 year 14 times higher than the stable class. Age and sex were associated with baseline frailty (eFI = 0.005/0.2 deficits per year of age, eFI = 0.016/0.6 deficits higher for women) across classes, but were only weakly related to class membership (all ORs approximately 1.0).

**Fig. 1:**
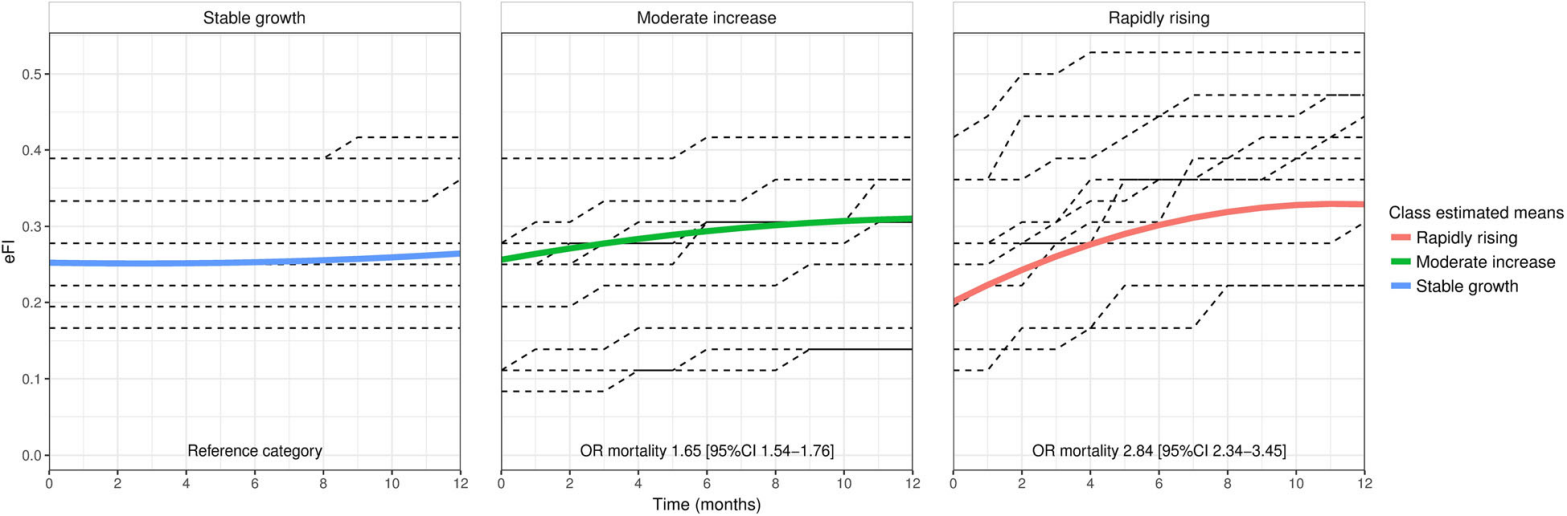
Estimated mean trajectories of eFI over 1 year for each of the three latent classes with a random sample of observed individual trajectories for each class

Compared with the ‘stable’ class both the ‘rapidly rising’ class (OR 2.84, 95% CI 2.34–3.45) and the ‘moderate growth’ class (OR 1.65, 95% CI 1.54–1.76) were found to be associated with mortality. Figure 1 displays the mean and observed individual trajectories for each class and the association between class and mortality risk.

To understand the population characteristics, the posterior probabilities for being in each class were reweighted to reflect the general population. Compared with the ‘stable class’, both the ‘rapidly rising’ class (OR 3.02, 95% CI 2.49–3.65) and the ‘moderate growth’ class (OR 1.58, 95% CI 1.49–1.67) were associated with an increased chance of mortality. Table 3 contains demographic information for the members of each class. Reweighting the controls to the population suggests that 1.1% of the population exhibited rapidly rising frailty; using this group to predict mortality within the year was highly specific (99.1% specificity), with 19.8% positive predictive value and 93.3% negative predictive value, but with low sensitivity (3.2%). Addition of the ‘moderately increasing’ class increased the sensitivity (27.8%) and negative predictive value (93.9%) at the expense of a decreased specificity (82.3%) and positive predictive value (10.4%).

**Table 3:** Demographic characteristics of the classes and the proportion of individuals in each class from the age- and sex-adjusted model reweighted to the population level

	*n*	%	*n* Female	% Female	Reweighted *n*^a^	%	Mean age	SD	Mean eFI^b^
Total	26,298	–	14,620	55.59	191,584	–	–	–	–
Rapidly rising	583	2.22	327	56.09	2100	1.10	85.81	5.80	0.21
Moderate growth	5571	21.18	3105	55.74	33,225	17.34	85.57	5.88	0.26
Stable	20,144	76.60	11,188	55.54	156,259	81.56	85.34	6.02	0.25
Cases total	13,149	–	7310	55.59	13,149	6.86	–	–	–
Rapidly rising	415	3.16	241	58.07	415	3.16	85.13	5.92	0.23
Moderate growth	3242	24.66	1743	53.76	3242	24.66	85.21	5.90	0.28
Stable	9492	72.19	5326	56.11	9492	72.19	85.11	6.01	0.27
Controls total	13,149	–	7310	55.59	178,435	93.14	–	–	–
Rapidly rising	167	1.27	86	51.50	1685	0.94	87.45	5.17	0.18
Moderate growth	2330	17.72	1362	58.45	29,983	16.80	86.08	5.82	0.24
Stable	10,652	81.01	5862	55.03	146,767	82.25	85.54	6.01	0.23

^a^Posterior probabilities for being in each class were combined with Office of National Statistics-derived population weights to reweight classes to the population level

^b^ Age adjusted

## Discussion

We have identified a distinct frailty trajectory that is associated with an increased risk of mortality amongst older adults. Individuals with rapidly rising frailty (average monthly rise in eFI of 0.01 or accrual of 0.4 deficits) are twice as likely to die within 12 months as individuals with stable frailty. Individuals in this class experience an initial monthly increase in eFI of 0.02, prior to slowing around 8–9 months prior to death. This rapidly rising class represents a small but important proportion of the population who may not be recognised as being at high risk of death, as they started from a lower mean baseline eFI than the rest of the population [17]. The ‘moderately increasing frailty’ class are also at a greater risk of mortality, with an average rise of 0.005 in eFI per month associated with a 58% increase in mortality.

Our study demonstrated that, for a small proportion of the population, significant changes in frailty occur over 1 year. Individuals with lower levels of frailty who experience a rapid rise have a greater risk of death over 1 year than individuals with higher, but stable frailty.

The association between severity of frailty measured at a single time point and risk of mortality has been robustly demonstrated at the population level [23]. However, our previous work found that this association did not translate into a useful measure for predicting individual risk of mortality when frailty is measured at a single time point, even close to death [18]. Building on previous work that has examined the relationship between longitudinal change in frailty and mortality at yearly intervals [21, 24], we have been able to measure frailty changes at monthly intervals over the final year of life. Our findings suggest that some individuals are resilient to higher but stable levels of frailty, and that changes in frailty status may be a more useful indicator of mortality risk. The positive predictive value and sensitivity for the rapidly rising trajectory mean that recognition of this trajectory alone may not be a reliable predictor of imminent death. However, it may be useful to support clinical judgement and as a risk stratification tool, especially as many individuals who follow this trajectory may not be considered as ‘at risk’ because of their low levels of baseline frailty. The Q Mortality risk prediction algorithm, which incorporates a frailty index and was designed specifically to assess mortality risk, has better cross-sectional prediction of mortality than the eFI. However, the frailty index implemented in Q mortality does not currently include a weighting for risk based on changes in frailty scores over time [25].

### Strengths and limitations

We have presented a large population-based longitudinal study of change in frailty measured with the eFI at monthly intervals. Our analysis used a measure of frailty that is available to the majority of general practitioners in England, enhancing the potential for the findings to be applied in practice. The trajectories have potential relevance to older populations in other countries, particularly when frailty is measured using a cumulative deficit approach. Much previously published work on frailty has used data from cohort studies, subject to all the potential limitations of observational research. In this study, participants were drawn from a large, unselected primary care cohort, demographically and geographically representative of England’s population, and data were routinely collected.

However, there were limitations to our approach. First, the design as implemented at data extraction meant controls were identified at the end of the study (1 January 2016), then matched to individuals who had died in the preceding year (matching was at date of death). Follow-up for the controls was determined from 1 January 2015 to 1 January 2016, but for the cases, follow-up was calculated for 1 year prior to date of death. Controls were not chosen at the point of case death, so this created an imbalance in ages between cases and controls whereby controls were, on average, 6 months older than cases. However, all analyses were adjusted for age, and as frailty increases with age, controls are more likely to have been measured at a higher point in their frailty trajectory and therefore would attenuate our findings if any residual effect of age remained.

Secondly, because study exclusion criteria were applied by ResearchOne at the time of data extraction, we do not have information about the number of people who were not eligible for inclusion in our analysis. Electronic health record systems also rely on clinicians recording their observations correctly using the Read code system, and we were unable to make any assessment of patterns of incorrect or differential coding; nevertheless, this is the same information as used for clinical decisions.

Finally, the way in which eFI is calculated and the data on which it is based (mostly long-term or irreversible deficits), means that eFI scores can only increase or remain stable. This means that any analysis using eFI would not be able to capture improvement or recovery in frailty, which is known to be feasible with interventions such as physical exercise [26–28].

## Conclusions

This longitudinal population-based study demonstrates that it is possible to use a frailty index calculated within electronic healthcare records to identify people who are at a higher risk of dying within 1 year. This has potential application in health services to support clinicians in identifying older adults dying with frailty who may have been overlooked by traditional approaches and to help ensure appropriate care is offered. The rapid initial rise in eFI from a low baseline, followed by a plateau, may be particularly useful in primary care, promoting recognition of people at risk of death, but also allowing time for intervention.

## Additional file

Additional file 1: **Table S1.** Model development latent growth curve model fit and parameters to determine model shape*. **Table S2.** Model description and goodness-of-fit statistics for latent growth curves including age and sex*. **Table S3.** Full description of latent growth mixture parameter characteristics and model fits*. (DOCX 41 kb)

## Abbreviations

BIC: Bayesian information criterion
CI: Confidence interval
eFI: Electronic frailty index
GP: General practitioner
NHS: National Health Service
OR: Odds ratio
SD: Standard deviation
UK: United Kingdom
