# Supplementary material for: Frailty trajectories to identify end of life: a longitudinal population-based study
Source: BMC Med. 2018 Sep 21;16:171. doi: 10.1186/s12916-018-1148-x (PMC6148780; doi:10.1186/s12916-018-1148-x)
Supplement: Supplementary file 1 — Table S1. Model development latent growth curve model fit and parameters to determine model shape*. Table S2. Model description and goodness-of-fit statistics for latent growth curves including age and sex*. Table S3. Full description of latent growth mixture parameter characteristics and model fits*. (DOCX 41 kb) [file 12916_2018_1148_MOESM1_ESM.docx]

## Table S1: Model development latent growth curve model fit and parameters to determine model shape*

| Model description and goodness of fit | | |  | Model parameter descriptions | | | | | | | | | | | | | | | | | |
| --- | --- | --- | --- | --- | --- | --- | --- | --- | --- | --- | --- | --- | --- | --- | --- | --- | --- | --- | --- | --- | --- |
| model description | Log likelihood | Adjusted BIC |  | Intercept mean | 95%CI | Intercept variance | 95%CI | Slope mean | 95%CI | Slope variance | 95%CI | Quadratic mean | 95%CI | Quadratic variance | 95%CI | Intercept-slope covariance | 95%CI | Intercept-quad covariance | 95%CI | Quad-slope covariance | 95%CI |
| Null model (Fixed intercept, no slope) | -1319450.28 | 2638998.56 |  | 26.253 | 26.214 - 26.292 | - | - | - | - | - | - | - | - | - | - | - | - | - | - | - | - |
| Intercept variance freed, no slope term | -679136.618 | 1358378.23 |  | 26.196 | 26.058 - 26.334 | 129.931 | 127.708 - 132.153 | - | - | - | - | - | - | - | - | - | - | - | - | - | - |
| Intercept variance free, fixed slope term | -648401.253 | 1296914.5 |  | 25.103 | 24.965 - 25.241 | 129.832 | 127.611 - 132.053 | 0.190 | 0.188 - 0.191 | - | - | - | - | - | - | - | - | - | - | - | - |
| Intercept variance free, slope variance free | -560321.393 | 1120768.77 |  | 25.122 | 24.985 - 25.259 | 128.407 | 126.209 - 130.604 | 0.189 | 0.185 - 0.192 | 0.080 | 0.079 - 0.082 | - | - | - | - | -0.131 | -0.171 - -0.091 | - | - | - | - |
| **Intercept variance free, slope variance free, quadratic term added** | **-492715.23** | **1060816.1** |  | **25.185** | **25.048 - 25.322** | **127.529** | **125.346 - 129.712** | **0.151** | **0.144 - 0.158** | **0.278** | **0.273 - 0.284** | **0.003** | **0.003 - 0.004** | **0.002** | **0.002 - 0.002** | **-0.109** | **-0.185 - -0.032** | **0.000** | **-0.005 - 0.006** | **-0.018** | **-0.018 - -0.017** |

*models used eFI multiplied by 100 to aid estimation and interpretation. Text in results refers to eFI in original units

Table S2: Model description and goodness of fit statistics for latent growth curves including age and sex*

| Model description | | |  |  | Model parameter characteristics | | | | | | | | | | | | | | | | |
| --- | --- | --- | --- | --- | --- | --- | --- | --- | --- | --- | --- | --- | --- | --- | --- | --- | --- | --- | --- | --- | --- |
| Covariates | Log likelihood | aBIC |  | Mean intercept | 95%CI | Mean slope | 95%CI | Mean quadratic | 95%CI | Age intercept | 95%CI | Age slope | 95%CI | Age quadratic | 95%CI | Sex intercept | 95%CI | Sex slope | 95%CI | Sex quadratic | 95%CI |
| No covariates | -530331.07 | 1060816.13 |  | 25.185 | 25.048 - 25.322 | 0.151 | 0.144 - 0.158 | 0.003 | 0.003 - 0.004 | - | - | - | - | - | - | - | - | - | - | - | - |
| Sex on intercept | -530149.23 | 1060459.44 |  | 26.368 | 26.186 - 26.550 | 0.151 | 0.144 - 0.158 | 0.003 | 0.003 - 0.004 | - | - | - | - | - | - | -2.664 | -2.937 - -2.391 | - |  | - |  |
| Sex on intercept and slope | -530146.5 | 1060467.98 |  | 26.368 | 26.186 - 26.550 | 0.156 | 0.146 - 0.165 | 0.003 | 0.002 - 0.004 | - | - | - | - | - | - | -2.665 | -2.938 - -2.392 | -0.010 | -0.023 - 0.004 | 0.001 | 0.000 - 0.002 |
| Age on intercept | -529407.1 | 1058975.17 |  | 25.185 | 25.053 - 25.317 | 0.151 | 0.144 - 0.158 | 0.003 | 0.003 - 0.004 | 0.492 | 0.470 - 0.514 | - |  | - | - | - | - | - | - | - | - |
| Age on Intercept and slope | -529400.12 | 1058975.23 |  | 25.185 | 25.053 - 25.317 | 0.151 | 0.144 - 0.158 | 0.003 | 0.003 - 0.004 | 0.490 | 0.468 - 0.512 | 0.002 | 0.001 - 0.003 | 0.000 | 0.000-0.000 | - | - | - | - | - | - |
| **Age and sex on intercept** | **-529340.06** | **1058848.1** |  | **25.893** | **25.715 - 26.071** | **0.15** | **0.144 - 0.158** | **0.003** | **0.003 - 0.004** | **0.467** | **0.444 - 0.489** | **-** | **-** | - | **-** | **-1.594** | **-1.864 - -1.325** | - | - | - | - |
| Sex on intercept age on intercept and slope | -529333.09 | 1058848.16 |  | 25.893 | 25.715 - 26.071 | 0.151 | 0.144 - 0.158 | 0.003 | 0.003 - 0.004 | 0.465 | 0.442 - 0.487 | 0.002 | 0.001 - 0.003 | 0.000 | 0.000-0.000 | -1.594 | -1.864 - -1.325 | - | - | - | - |
| Sex on intercept and slope age on intercept | -529337.33 | 1058856.65 |  | 25.893 | 25.715 - 26.071 | 0.156 | 0.144 - 0.158 | 0.003 | 0.002 - 0.004 | 0.467 | 0.444 - 0.489 | - |  | - |  | -1.595 | -1.865 - -1.325 | -0.010 | -0.023 - 0.004 | 0.001 | 0.000 - 0.002 |
| Age and sex on intercept and slope | -529330.13 | 1058856.23 |  | 25.895 | 25.717 - 26.073 | 0.154 | 0.145 - 0.163 | 0.003 | 0.002 - 0.004 | 0.465 | 0.442 - 0.487 | 0.002 | 0.001 - 0.003 | 0 | 0.000-0.000 | -1.600 | -1.869 - -1.330 | -0.01 | -0.020 - 0.008 | 0.001 | 0.000 - 0.002 |

*models used eFI multiplied by 100 to aid estimation and interpretation. Text in results refers to eFI in original units

Table S3: Full description of latent growth mixture parameter characteristics and model fits*

| Model fit statistics | | | |
| --- | --- | --- | --- |
| n classes | LL0 | aBIC | entropy |
| 1 | -529340.064 | 1058848.111 | - |
|  |  |  |  |
| 2 | -523683.502 | 1047576.981 | 0.971 |
|  |  |  |  |
|  |  |  |  |
|  |  |  |  |
| **3** | **-519497.615** | **1039247.203** | **0.965** |
|  |  |  |  |
|  |  |  |  |
|  |  |  |  |
| 4 | 512368.968 | 1025031.905 | 0.995 |
|  |  |  |  |
|  |  |  |  |
|  |  |  |  |

| Model description | | | | | | | | | | | | | | | | | | | | | | |  |
| --- | --- | --- | --- | --- | --- | --- | --- | --- | --- | --- | --- | --- | --- | --- | --- | --- | --- | --- | --- | --- | --- | --- | --- |
| class | N in class | % in class | I ON age | 95%CI | OR class on age | 95%CI | I ON male | 95%CI | OR class on male | 95%CI | Mean intercept | 95%CI | Mean slope | 95%CI | Mean quadratic | 95%CI | Intercept variance | 95%CI | Slope variance | 95%CI | Quadratic variance | 95%CI | |
| 1 | 26298 | 100.00% | 0.467 | 0.444 - 0.489 | - | - | -1.594 | -1.863 - -1.326 | - |  | 25.893 | 25.712 - 26.074 | 0.151 | 0.145 - 0.158 | 0.003 | 0.003 - 0.004 | 118.328 | 116.256 - 120.401 | 0.278 | 0.266 - 0.291 | 0.002 | 0.000 - 0.002 | |
|  |  |  |  |  |  |  |  |  |  |  |  |  |  |  |  |  |  |  |  |  |  |  | |
| 1 | 24566 | 93.41% | 0.467 | 0.445 - 0.490 | ref | - | -1.592 | -1.860 - -1.324 | ref | - | 26.075 | 25.890 - 26.259 | 0.047 | 0.042 - 0.052 | 0.008 | 0.008 - 0.009 | 117.886 | 115.823 - 119.948 | 0.129 | 0.123 - 0.135 | 0.001 | 0.001 - 0.001 | |
| 2 | 1732 | 6.59% | 0.467 | 0.445 - 0.490 | 1.015 | 1.006 - 1.023 | -1.592 | -1.860 - -1.324 | 1.022 | 0.917 - 1.128 | 23.36 | 22.788 - 23.932 | 1.600 | 1.531 - 1.669 | -0.066 | -0.072 - -0.061 | 117.886 | 115.823 - 119.948 | 0.129 | 0.123 - 0.135 | 0.001 | 0.001 - 0.001 | |
|  |  |  |  |  |  |  |  |  |  |  |  |  |  |  |  |  |  |  |  |  |  |  | |
|  |  |  |  |  |  |  |  |  |  |  |  |  |  |  |  |  |  |  |  |  |  |  | |
| **1** | **20144** | **76.60%** | **0.466** | **0.444 - 0.489** | **ref** | **-** | **-1.598** | **-1.866 - -1.329** | **ref** | **-** | **25.959** | **25.763 - 26.155** | **-0.080** | **-0.085 - -0.074** | **0.015** | **0.015 - 0.015** | **117.742** | **115.676 - 119.808** | **0.058** | **0.053 - 0.06.** | **0.001** | **0.001 - 0.001** | |
| **2** | **5572** | **21.19%** | **0.466** | **0.444 - 0.489** | **1.007** | **1.002 - 1.012** | **-1.598** | **-1.866 - -1.329** | **1.015** | **0.951 - 1.082** | **26.232** | **25.907 - 26.558** | **0.802** | **0.765 - 0.838** | **-0.029** | **-0.032 - -0.027** | **117.742** | **115.676 - 119.808** | **0.058** | **0.053 - 0.06.** | **0.001** | **0.001 - 0.001** | |
| **3** | **582** | **2.21%** | **0.466** | **0.444 - 0.489** | **1.014** | **1.000 - 1.028** | **-1.598** | **-1.866 - -1.329** | **0.999** | **0.840 - 1.189** | **20.583** | **19.387 - 21.779** | **2.294** | **2.115 - 2.472** | **-0.102** | **-0.114 - -0.09** | **117.742** | **115.676 - 119.808** | **0.058** | **0.053 - 0.06.** | **0.001** | **0.001 - 0.001** | |
|  |  |  |  |  |  |  |  |  |  |  |  |  |  |  |  |  |  |  |  |  |  |  | |
| 1 | 19943 | 75.84% | 0.467 | 0.444 - 0.468 | ref | - | -1.594 | -1.863 - -1.326 | ref | - | 25.989 | 25.792 - 26.186 | -0.095 | -0.099 - -0.092 | 0.02 | 0.015 - 0.016 | 117.801 | 115.735 - 119.866 | 0.044 | 0.041 - 0.047 | 0.001 | 0.001 - 0.001 | |
| 2 | 4784 | 18.19% | 0.467 | 0.444 - 0.468 | 1.006 | 1.000 - 1.083 | -1.594 | -1.863 - -1.326 | 1.021 | 0.956 - 1.011 | 26.410 | 26.084 - 26.737 | 0.648 | 0.631 - 0.664 | -0.02 | -0.022 - 0.018 | 117.801 | 115.735 - 119.866 | 0.044 | 0.041 - 0.047 | 0.001 | 0.001 - 0.001 | |
| 3 | 1232 | 4.69% | 0.467 | 0.444 - 0.468 | 1.012 | 1.008 - 1.015 | -1.594 | -1.863 - -1.326 | 1.010 | 0.968 - 1.061 | 24.057 | 23.421 - 24.693 | 1.498 | 1.447 - 1.548 | -0.063 | -0.067 - -0.059 | 117.801 | 115.735 - 119.866 | 0.044 | 0.041 - 0.047 | 0.001 | 0.001 - 0.001 | |
| 4 | 339 | 1.29% | 0.467 | 0.444 - 0.468 | 1.021 | 1.009 - 1.034 | -1.594 | -1.863 - -1.326 | 1.036 | 0.916 - 1.188 | 19.320 | 18..852 - 20.788 | 2.836 | 2.620 - 3.016 | -0.14 | -0.155 - -0.126 | 117.801 | 115.735 - 119.866 | 0.044 | 0.041 - 0.047 | 0.001 | 0.001 - 0.001 | |

*models used eFI multiplied by 100 to aid estimation and interpretation. Text in results refers to eFI in original units
